# Investigation of Biomarker Response to SGLT2 Inhibition in Heart Failure *(SiN-HF)*

**DOI:** 10.1007/s10557-025-07800-3

**Published:** 2025-12-05

**Authors:** Patrick Savage, Katie Linden, Lana Dixon, David Grieve, Chris Watson

**Affiliations:** 1 Royal Victoria Hospital Cardiology department, Belfast, Northern Ireland; 2 Wellcome-Wolfson Institute for Experimental Medicine, Queen’s University, Belfast, Northern Ireland

**Keywords:** Heart failure, SGLT2 inhibitors, Biomarkers, Echocardiography

## Abstract

**Introduction:**

Following several landmark trials, sodium glucose co-transport (SGLT) 2 inhibitors, have been established as a guideline directed therapy for heart failure (HF). Moreover, their benefit has been established across the spectrum of left ventricular (LV) dysfunction. Recent evidence has implicated inflammatory, autophagic and anti-fibrotic pathways as potentially being associated with their treatment effects.

**Aim:**

We therefore sought to evaluate the patterns of biomarker response to SGLT2 and associations with myocardial remodelling and patient reported outcomes in heart failure.

**Methods:**

This was a 26-week, single-arm prospective evaluation of the effects of SGLT2 inhibition on the patterns of novel cardiac biomarker response to SGLT2 inhibition and associations with myocardial remodeling and patient reported outcomes in patients with heart failure. Baseline echocardiography, serum analysis (standard care and novel biomarkers) and quality of life (QoL) metrics were assessed prior to SGLT2i therapy and at 26-week follow-up. Novel biomarkers were analysed using enzyme-linked immunosorbent assays. Data were analysed using SPSS (IBM SPSS Statistics, Version 28.0). Clinical Trials.gov identifier: *NCT06140251*.

**Results:**

Forty-six patients were recruited with forty patients undergoing biomarker analysis (mean age 67.2+/-8.2 years: 30% female). Mean LV ejection fraction (LVEF) at baseline was 45.3+/9.8% (ischaemic aetiology: 40.0%, diabetic: 5%). At a median follow-up of 196 days, soluble suppression of tumorigenicity 2 (sSt2) fell significantly (mean difference − 13.5pg/ml [95% CI: -17.9 to − 8.9: *p* < 0.001]), with no significant change in interleukin (IL)1-β, IL-4, IL-6 or insulin growth factor binding protein 1 (IGFBP-1) (all p = ns). Interestingly, delta change in IL-6 modestly correlated with change in global longitudinal strain (GLS) (%) (*r*=0.43, *p* = 0.012). Change in GLS (%) was not correlated with other novel cardiac biomarkers.

**Conclusion:**

In our cohort treated with SGLT2i, we found a significant decrease in levels of sSt2, a protein implicated in cardiac fibrosis. We noted no change in circulating levels of IL-1β, IL-4, IL-6 or IGFBP1. Interestingly, when compared to changes in LV GLS (%), we observed that decreasing levels of IL-6 correlated with improved LV GLS (%). More exploration of these novel associations are warranted.

**Supplementary Information:**

The online version contains supplementary material available at 10.1007/s10557-025-07800-3.

## Introduction

Since the publication of the now landmark *EMPA-REG OUTCOME* (Empagliflozin Cardiovascular Outcome Event Trial in Type 2 Diabetes Mellitus Patients) study, nearly a decade ago, SGLT2 inhibitors have emerged as a new pillar of heart failure (HF) therapy. Unique to this drug class, their cardio-protective effects appear to extend across the spectrum of left ventricular (LV) dysfunction [1, 2]. Their place in current guideline HF therapy has been consolidated by multiple positive placebo-controlled Randomised Controlled Trials (RCT) and re-iterated in data from several robust meta-analyses [3–5]. More-over, their plethora of effects appear to extend beyond the realm of HF with incorporation into diabetic, renal and atherosclerotic cardiovascular disease (ASCVD) guidelines also [6–8].

Despite the ubiquity of positive RCT data, much remains unclear regarding their mechanisms of action [9]. The early hypothesis that their cardio-protective effects were mediated simply by glycosuria causing diuresis and CV risk reduction were soon refuted, once it was seen their effects occurred independently of baseline HbA1c levels [10]. In addition to mediating general CV risk reduction from weight loss and improved blood pressure control, several end-organ effects have been observed with SGLT2 inhibition, including haemoconcentration, erythropoiesis, natriuresis and improved myocardial energetics [11–13]. Indeed, cardiac imaging data has also demonstrated that SGLT2 inhibition has direct effects on reverse cardiac remodeling [14–16]. Although we can extrapolate from previous RCT data as to the mechanisms driving this remodeling, much remains unclear.

Of emerging interest, is the role of SGLT2 inhibition in modification of both inflammatory and fibrotic pathways in HF [17]. Both these processes are known to be involved in driving negative remodeling in HF and early pre-clinical data has implicated SGLT2 inhibition in modification of several of these pathways. Of particular interest, are several key pro-inflammatory cytokines such as interleukin (IL)−1β, IL-4 and IL-6 in addition to soluble suppression of tumorigenicity 2 (sSt2) and insulin growth factor binding protein 1 (IGFBP-1), which are involved in cardiac fibrosis [3, 18, 19].

High levels of IL-4 are associated with cardiac fibrosis and endothelial dysfunction and have been implicated in the development of atherosclerosis [20]. IL-4 induces oxidative stress mediators including cytokines, chemokines and several adhesion molecules, in addition to promoting cardiac fibrosis by production of cardiac monocyte chemoattractant protein 1 (MCP-1) and fibroblasts, in addition to activating Reactive Oxygen Species (ROS) mediated expression of the transcription factor activator protein (AP) 1 and collagen-1a in cardiac fibroblasts. IL-6 has been implicated in atherosclerosis, heart failure and stroke [21, 22]. Interestingly a meta-analysis of SGLT2 inhibitor use in diabetic patients demonstrated that lower levels of IL-6 are associated with SGLT2 inhibitor use, a finding also noted in early studies of patients with CKD [18, 23].

IL-1β is a key pro-inflammatory cytokine produced by activated macrophages which is a driver of multiple pro-inflammatory processes and has been implicated in the progression of atherosclerosis, HF and myocardial infarction. In several in vitro studies IL-1β expression has been shown to be suppressed by SGLT2 inhibition [24, 25].

With respect to cardiac fibrosis, sSt2 is a protein secreted in response to activation of myocardial stretch and is known to stimulate cardiac fibrosis and hypertrophy with high circulating levels seen in patients with heart failure [26]. With respect to SGLT2 inhibition in heart failure, no clinical studies have been conducted to date exploring its relationship to sSt2, highlighting its novelty as a potential mechanistic pathway. Additionally, IGFBP-1, a protein which binds to and inhibits IGF-1 and is implicated in positive cardiac remodeling, has been highlighted as another possible protein of interest within a proteomic sub-study of the EMPORER trials [27].

Therefore, the purpose of this study was to evaluate the impact of SGLT2 inhibition on the patterns of novel cardiac biomarker response in patients with HF, in addition to evaluating its association with reverse cardiac remodeling as determined by echocardiography. Secondly, we sought to evaluate the effects of SGLT2 inhibition on standard of care biomarkers in addition to quality-of-life outcomes (QoL)’s.

## Methods

### Study Design

This was a 26-week, open label, single-arm prospective evaluation of the effects of SGLT2 inhibition on the patterns of novel cardiac biomarker response to SGLT2 inhibition and associations with myocardial remodelling and patient reported outcomes in patients with heart failure. The trial protocol, consent, recruitment procedure and enrolment were approved by the UK Research Ethics Committee with local governance approval, the full details of which are available in the (Supplement).

In brief, adult patients with stable symptoms and otherwise on optimal medical therapy who were eligible for commencement on a SGLT2 inhibitor for the treatment of heart failure as per standard care guidelines were considered. The diagnosis of HF was confirmed with recent echocardiography < 1 year with a left ventricular ejection fraction (LVEF) < 50% on echocardiography, or if > 50% LVEF with objective echocardiographic evidence of cardiac dysfunction (left atrial [LA) volume index > 34 ml/m2, E/e’ ratio > 9, tricuspid regurgitation [TR] velocity > 2.8 m/s, pulmonary arterial systolic pressure [PASP] > 25mmHg or left ventricular hypertrophy [LVH]) as outlined in current ESC Heart failure guidance.

Key exclusion criteria included: hospitalisation for heart failure within 4 weeks prior to enrolment, EGFR < 25 mL/min/1.73m2 at screening, type 1 diabetes, suspected cardiac amyloid, myo- or pericarditis or infiltrative cardiomyopathy.

Patients identified with heart failure (both reduced ejection fraction and preserved), on otherwise optimally tolerated standard therapy and were candidates for treatment with SGLT2 inhibition were identified from a local heart failure database, and local heart failure clinics. Following signed, informed consent and screening, patients underwent baseline assessment including clinical evaluation, completion of KCCQ-12 score, biomarker sampling and echocardiography, followed by commencement of a SGLT2 inhibitor as per standard care. The SGLT2 inhibitor used was at the discretion of the prescribing clinician. At 26 weeks these data points were repeated. The study was conducted in accordance with Good Clinical Practice and the Declaration of Helsinki and is registered as Clinical Trials.gov identifier: ***NCT06140251.***

## Endpoints

The full study objectives and endpoints are detailed in Table 1. In brief, the *primary study outcome* evaluated whether SGLT2 inhibition in heart failure affects changes in novel cardiac biomarkers. This was an exploratory evaluation of novel cardiac pathways which may serve to establish, as of yet unknown, patterns of novel biomarker response to SGLT2 inhibition in heart failure.

**Table 1:** Table demonstrating study objectives and associated endpoints

Primary and secondary study objectives and endpoints	
Primary Objectives	Study Objective
To evaluate novel mechanistic pathways relating to SGLT2 inhibition in heart failure.	Change in levels of novel biomarkers of interest including sST2 (pg/ml), IGFBP1 (µg/ml), IL1β (pg/ml), IL-4 (pig/ml) and IL-6 (pg/ml), from baseline to 26 weeks.
Secondary Objectives	
To evaluate if changes in standard cardiac biomarkers following SGLT2 inhibition in heart failure correlate with quantitative and qualitative measures of heart outcomes.	Correlation of change in LV GLS (%), LVEF (%), LVESVi (mls/m^2^), LVEDVi (mls/m^2^), LAVi (mls/m^2^), with change in novel and standard care biomarkers. Change in NT-proBNP (ng/L) and hs-TnT (ng/L) from baseline to 26 weeks.
Endpoint	Correlation of change of QoL outcomes with standard and novel biomarkers following from baseline to 26 weeks.

*sST2* soluble suppression of tumorigenicity 2 protein, *IGFBP1* Insulin-like growth factor-binding protein, *IL-1B* Interleukin-1 beta, *IL-4* Interleukin-4, *IL-6* Interleukin-6, *CRP* C-reactive protein, *HbA1c* glycosylated haemoglobin, *SGLT2i* Sodium-glucose co-transport 2 inhibitor, *GLS* Global longitudinal strain, *LVEF* Left ventricular ejection fraction, *LVESVi* Left ventricular end systolic volume index, *LVEDVi* Left ventricular end diastolic volume index, *LAVi* Left atrial volume index

*Secondary outcomes* evaluate changes in standard of care biomarkers in response to SGLT2 inhibition in heart failure. Additionally, we sought to evaluate changes in echocardiographic parameters and quality of life (QoL) heart failure outcomes following SGLT2 inhibitor therapy and evaluate the relationship between these clinical parameters and novel and standard care biomarkers.

## Novel Biomarker Analysis

As part of biomarker evaluation, peripheral venous blood sampling was performed prior to SGLT2 inhibitor commencement and at follow-up, with the samples subsequently centrifuged at 2500 g for 10 min with subsequent aliquoting and storage − 80 ∘C. Enzyme-linked immunosorbent assay (ELISA) analysis was performed to quantify serum levels of proteins of interest, specifically sST2, IGFBP-1, IL-β, IL-4 and IL-6. All assays were performed using assay kits supplied by R&D systems and were performed according to the manufacturer’s instructions.

## Echocardiography

Echocardiography was performed at baseline and at follow-up by a British Society Echocardiography (BSE) accredited operator to include the full standard BSE dataset with averages of measurements taken on sequential cardiac cycles. All images were obtained using a Phillips EPIC™ CVX model echocardiography machine.

Left ventricular internal diameter at end diastole (LVIDd) and end systole (LVIDs), interventricular septum diameter (IVSd) and left ventricular posterior wall thickness in diastole (LVPWd) measurements will be obtained in the parasternal long axis (PSAX) views. Left atrial (LA) volume, left ventricular volumes and LVEF will be calculated using biplane volumes and indexed to body surface area (BSA). Where image quality is sub-optimal for Simpson’s biplane assessment of LVEF, a visual estimate will be given.

The maximum velocity of early filling (E-wave) during diastole (EVmax), ratio of early ventricular diastolic (E-wave) and atrial filling (A-wave), and deceleration time of the mitral E-wave (DT), will be measured in the apical four chamber view (A4C) using pulsed wave doppler at the level of the mitral valve (MV) leaflet tips at end-expiration. Additionally, the velocity of early myocardial relaxation (e′), velocity of myocardial tissue during ventricular systole (s’) and E to early diastolic mitral annular tissue velocity (E/e′) will be measured in the A4C view using tissue doppler imaging. Global two-dimensional speckle tracking will be performed in the two, three and four chamber views to obtain values for global longitudinal strain (GLS). The endocardial border will be traced automatically along the region of interest at end systole and adjusted as appropriate. Peak GLS will be calculated as an average of the peak strain from the three projections. In the case of sub-optimal image quality, the data will be excluded from the final analysis.

### Statistical Analysis

Continuous variables are expressed as mean+/-SD with categorical variable expressed as *n* (%), unless otherwise stated. Normality was assessed using the Shapiro–Wilk test and visually assessed using quantile plots. Differences in categorical data were assessed using a X^2^ test and differences between groups for continuous data assessed using a two-tailed paired student t-test if normally distributed and Wilcoxon signed-rank test if non-normally distributed. Bivariate correlation was assessed using Pearson linear correlation or Spearman rank correlation, if non-normally distributed. Analysis performed using SPSS V28.0 (IBM). Full details of statistical methods and power calculations for this study are provided in the Supplement.

## Results

### Demographics

**Fig. 1:**
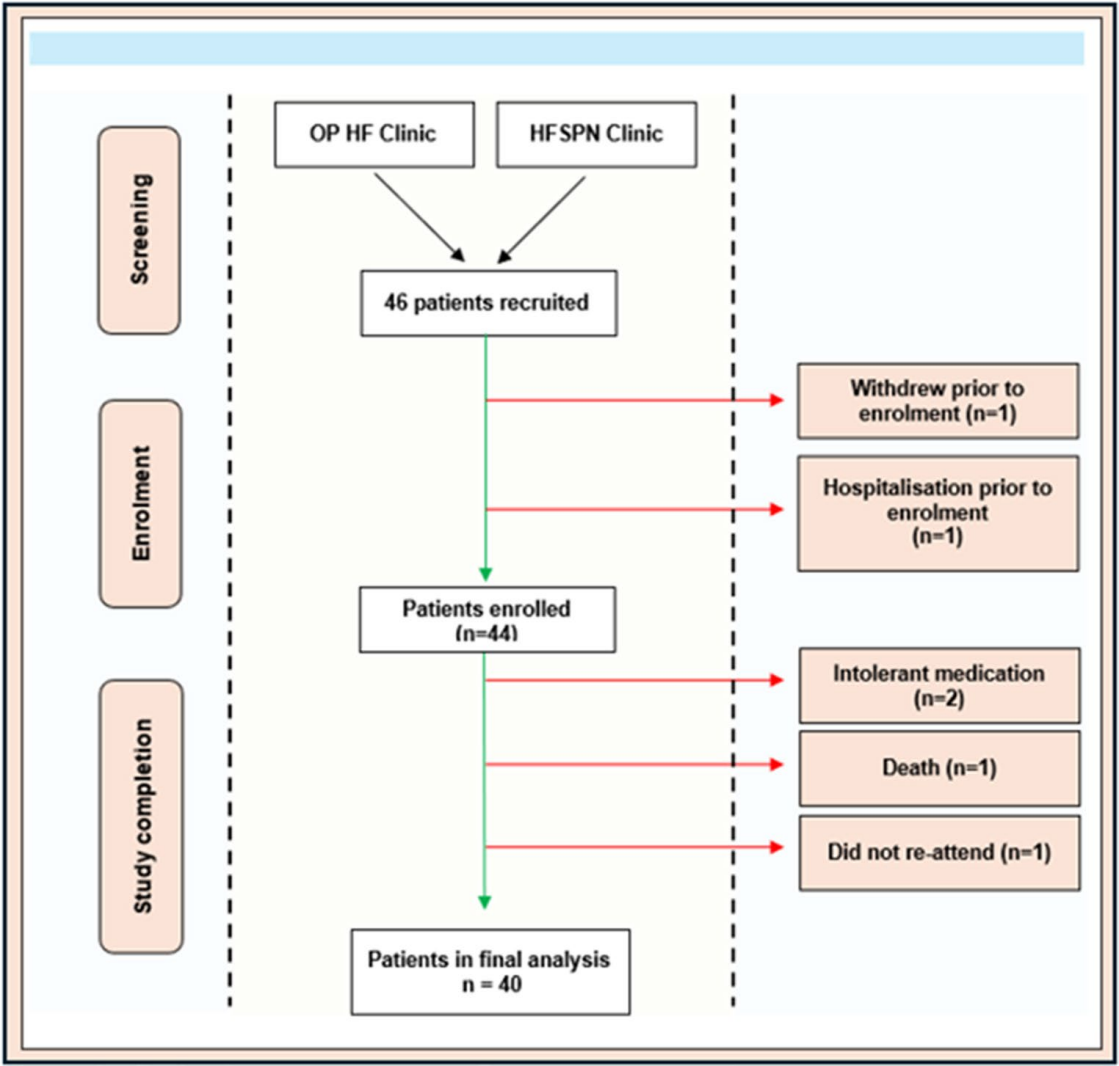
Flowchart demonstrating recruitment and enrolment. Of the 46 patients recruited, 44 were enrolled in the final study. Of these, two were intolerant of SGLT2 inhibitor therapy, and one patient did not return for follow-up echocardiography. There was one patient death

A total of 46 patients meeting the study inclusion criteria were enrolled in the study, with 40 patients included in the final analysis. Full recruitment details are depicted in Fig. 1. At enrolment, the mean age was 67.2+/−8.2 years (30% female) and mean LVEF was 45.3+/−9.8 %. The majority of patients were non-diabetic (95%) with a mean NYHA score of 2.4+/-0.5 and a median duration from initial HF diagnosis of 74.1 months (36.4 to 115.7) (Table 2). All patients received SGLT2i with dapagliflozin (10 mg OD) with a median duration of follow-up following initiation of 6.8 months. A full description of baseline clinical parameters and QoL metrics are available in the Appendix (A1) along with concurrent cardiac pharmacotherapy (at baseline and follow-up).

**Table 2:** Baseline demographics of study population

Baseline Demographics		
		Number (n, %)
Age (yrs)		67.2+/-8.2
Total patients		40 (100.0)
Gender	Male	28 (70.0)
	Female	12 (30.0)
Ethnicity		
	White	40 (100.0)
Comorbidities		
	Hypertension	22 (55.0)
	Chronic kidney disease	15 (37.5)
	Hypercholesterolaemia	13 (32.5)
	Myocardial infarction	16 (40.0)
	COPD	8 (20.0)
	Autoimmune disease	1 (2.5)
	Type 2 DM	2 (5.0)
	Atrial fibrillation	12 (30.0)
Aetiology HF		
	Ischaemic	16 (40.0)
	Non-ischaemic	24 (60.0)
Smoking history		6 (15.0)
Implantable cardiac device		8 (20.0)
Left ventricular ejection fraction (%)		45.3+/-9.8
NYHA score		2.5+/-0.5
Time from HF diagnosis (months)		74.1 (36.4 to 115.7) *
BMI (kg/m2)		27.5+/-6.1

Categorical data are presented n (%) with continuous data as mean+/- SD. Data marked by * are expressed as median (IQR)

*BMl* Body mass index, *COPD* Chronic obstructive pulmonary disease, *DM* Diabetes mellitus, *HF* Heart failure, *NYHA* New York Heart Association

## Novel Biomarker Analysis

The delta change in five novel biomarkers in response to SGLT2 inhibition were assessed in our population, these included IGFBP1, sSt2, IL-1β, IL-4 and IL-6 (Table 3). Of these, IL-4 was not detectable in our cohort.

**Table 3:** Change in novel biomarkers following six months SGLT2 inhibitor therapy

Change in novel biomarkers following SGLT2 inhibition					
Novel biomarkers	Detectable pairs *(n)*	Baseline	Follow-up	Mean difference (95% CI)	p value
IGFBP1 (µg/ml)	40	13.06 (2.2 to 16.9)	16.5 (4.0 to 26.9)	+3.4 (-8.0 to 1.2)	p = 0.141
sST2 (pg/ml)	40	40.4 (31.9 to 49.6)	26.9 (21.2 to 36.6)	-13.5 (-8.9 to -17.9)	**p<0.001**
IL1-β (pg/ml) *	15	0.34+/-0.19	0.56+/-0.41	+0.21 (-0.47 to +0.04)	p=0.09
IL4 (pg/ml)	0	-	-	-	-
IL6 (pg/ml)	40	4.8 (0.9 to 12.2)	6.4 (1.2 to 29.2)	+1.5 ( -3.4 to 0.3)	p=0.10

Data presented as mean (IQR) with differences in means assessed using a paired two tailed t-test with significance defined as p<0.05. *Data displayed as mean+/-SD

*SGLT2i* Sodium-glucose co-transport 2 inhibitor, *sST2* soluble suppression of tumorigenicity 2 protein, *IGFBP1* Insulin-like growth factor-binding protein, *IL-1B* Interleukin-1 beta, *IL-4* Interleukin-4, *IL-6* Interleukin-6

**Fig. 2:**
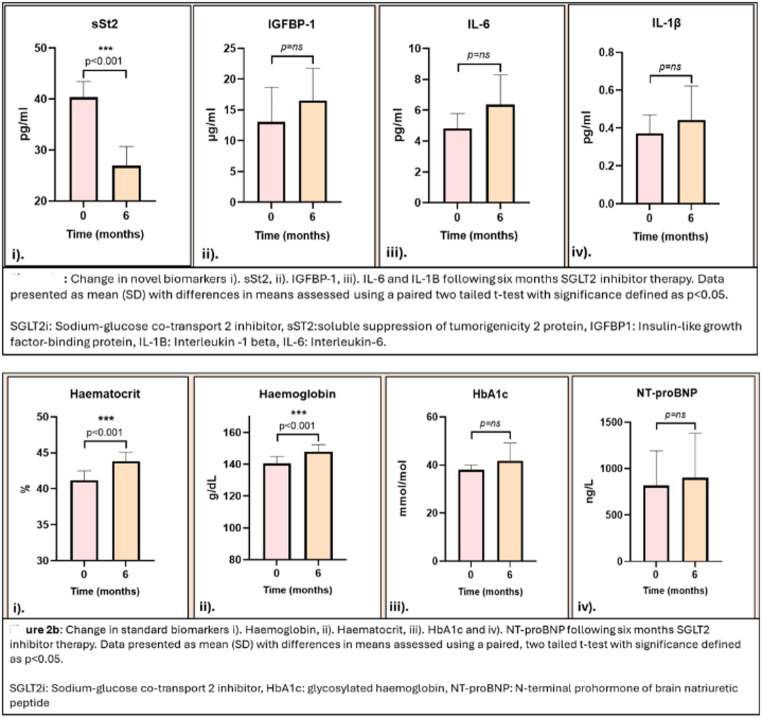
a). Change in novel biomarkers i). sSt2, ii). IGFBP-1, iii). IL-6 and IL-1B and b). change in standard biomarkers i). Haematocrit, ii). Haemoglobin, iii). HbA1c and iv). NT-proBNP following six-months SGLT2 inhibitor therapy. Data presented as mean (SD) with differences in means assessed using a paired, two tailed t-test with significance defined as p < 0.05. Sodium-glucose co-transport 2 inhibitor, sST2: soluble suppression of tumorigenicity 2 protein, IGFBP1: Insulin-like growth factor-binding protein, IL-1B: Interleukin − 1 beta, IL-6: Interleukin-6, HbA1c: glycosylated haemoglobin, NT-proBNP: N-terminal prohormone of brain natriuretic peptide

There was a significant decrease in levels of sSt2 following SGLT2 inhibition (mean difference − 13.5pg/mL (95% CI: −8.9 to −17.9; *p* < 0.001) however, there was no significant difference in IGFBP-1 (+ 3.5 µg/mL [95% CI: −8.0 to 1.2; *p* = 0.141]), IL-6 (+ 1.5pg/mL [95% CI: −3.4 to 0.3; *p* = 0.10]) or L-1β (+ 0.21 pg/ml [95% CI −0.47 to + 0.04: *p* = 0.09]) (Figure 2***a***). These results remained consistent when delineated by aetiology (ischaemic vs. non-ischaemic). The full details of these analyses are provided in the Appendix (A2).

### Biomarker Correlation with GLS (%)

All patients underwent baseline and follow-up echocardiographic assessment. Of these, 36 patients had windows sufficient for interval global longitudinal strain analysis. At follow-up, there was no significant difference in GLS (%) (13.9+/−3.8 % to 14.1+/−3.7 %, *p* = 0.803) following SGLT2 inhibition. Additionally, in our cohort, duration of therapy did not appear to be associated with degree of GLS % improvement (r −0.1, p = ns). A full description of echocardiographic findings are detailed in the Appendix (A3).

**Fig. 3:**
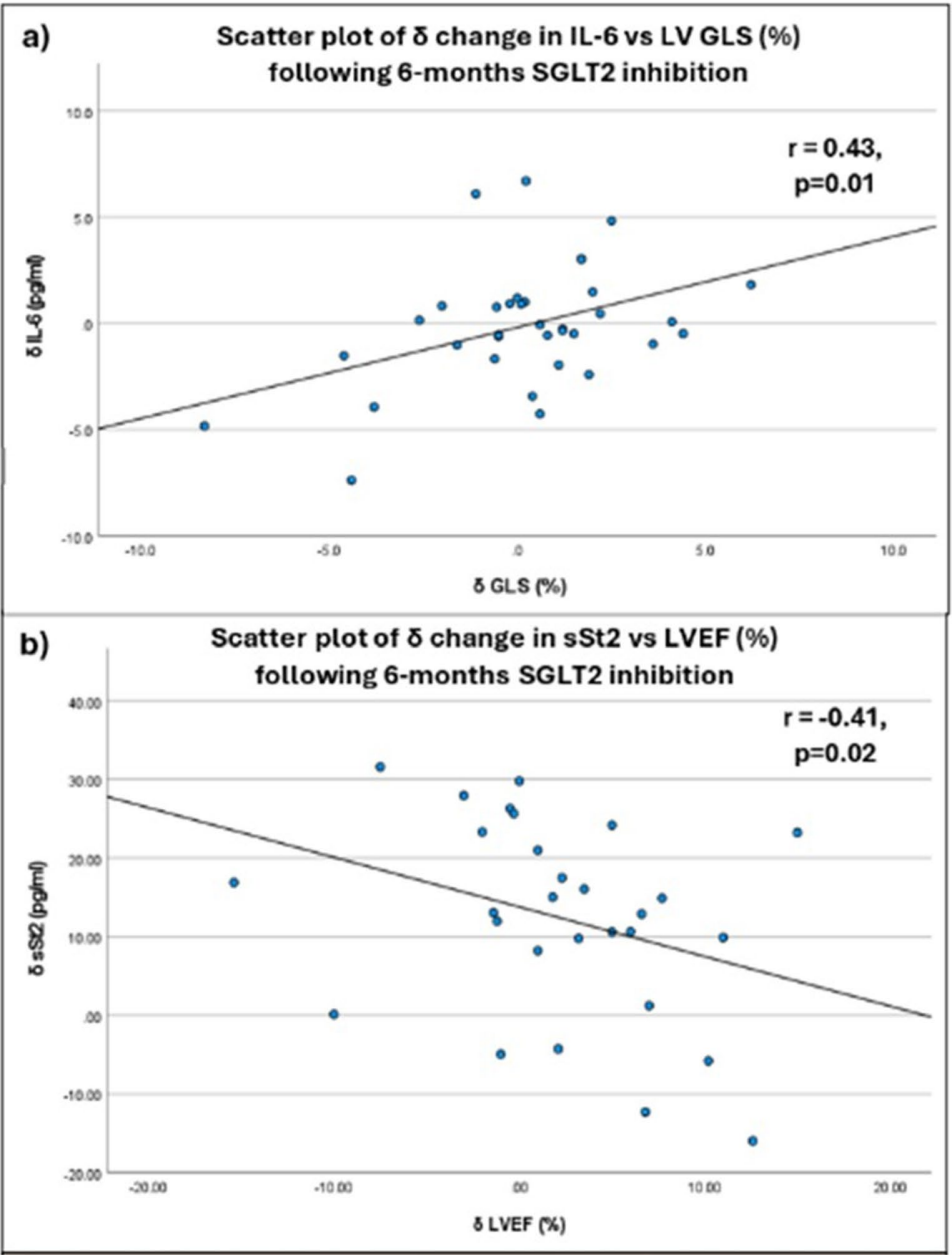
Scatter plots demonstrating correlation between a). delta of IL-6 vs. GLS (%) and b). sSt2 vs. LVEF (%). An improvement in GLS (-ve delta) was correlated with a decrease in IL-6. Improvement in LVEF % (+ ve delta) was associated with a fall in sSt2. Analysis performed using Pearson bivariate regression with a two-tailed p value. Significance defined as p < 0.05 GLS: Global longitudinal strain, LVEF: Left ventricular ejection fraction, SGLT2i: Sodium-glucose co-transport 2 inhibitor, sST2: soluble suppression of tumorigenicity 2 protein, IL-6: Interleukin-6

Of the novel biomarkers assessed, IL-6 reduction over time was noted to be modestly correlated with delta change in absolute GLS (%) (*r* = 0.43, *p* = 0.012) (Fig3a. ). No difference in demographics or comorbidities at baseline were noted between patients who did or did not experience reverse cardiac remodeling (+ RCR, defined as ≥ 10 % relative improvement in GLS%). Notably, patients who had + RCR had a lower LVEF at baseline (41.9 vs. 47.2 %, *p* = 0.02) (Appendix A4). In a further sub-analysis delineating by aetiology (ischaemic vs. non-ischaemic), we noted that this effect was consistent in patients with non-ischaemic CM (*r* = 0.507, *p* = 0.019) however was non-significant in those with ischaemic HF (*r* = 0.305, *p* = 0.289).

Interestingly, in patients who had a ≥ 10% relative improvement in GLS following SGLT2 inhibition, IL-6 was noted to fall (5.7+/−3.9 pg/ml to 5.0+/−2.9 pg/ml, *p* = 0.20) whereas in patients who did not see improvements in GLS, IL-6 levels rose (4.6+/−2.7 to 5.9+/−5.9, *p* = 0.2); however, neither of these trends reached statistical significance. Furthermore, change in GLS (%) was not correlated with other novel cardiac biomarkers tested following six months SGLT2 inhibitor therapy.

### Novel Biomarker Prediction of Improvement in GLS (%)

**Fig. 4:**
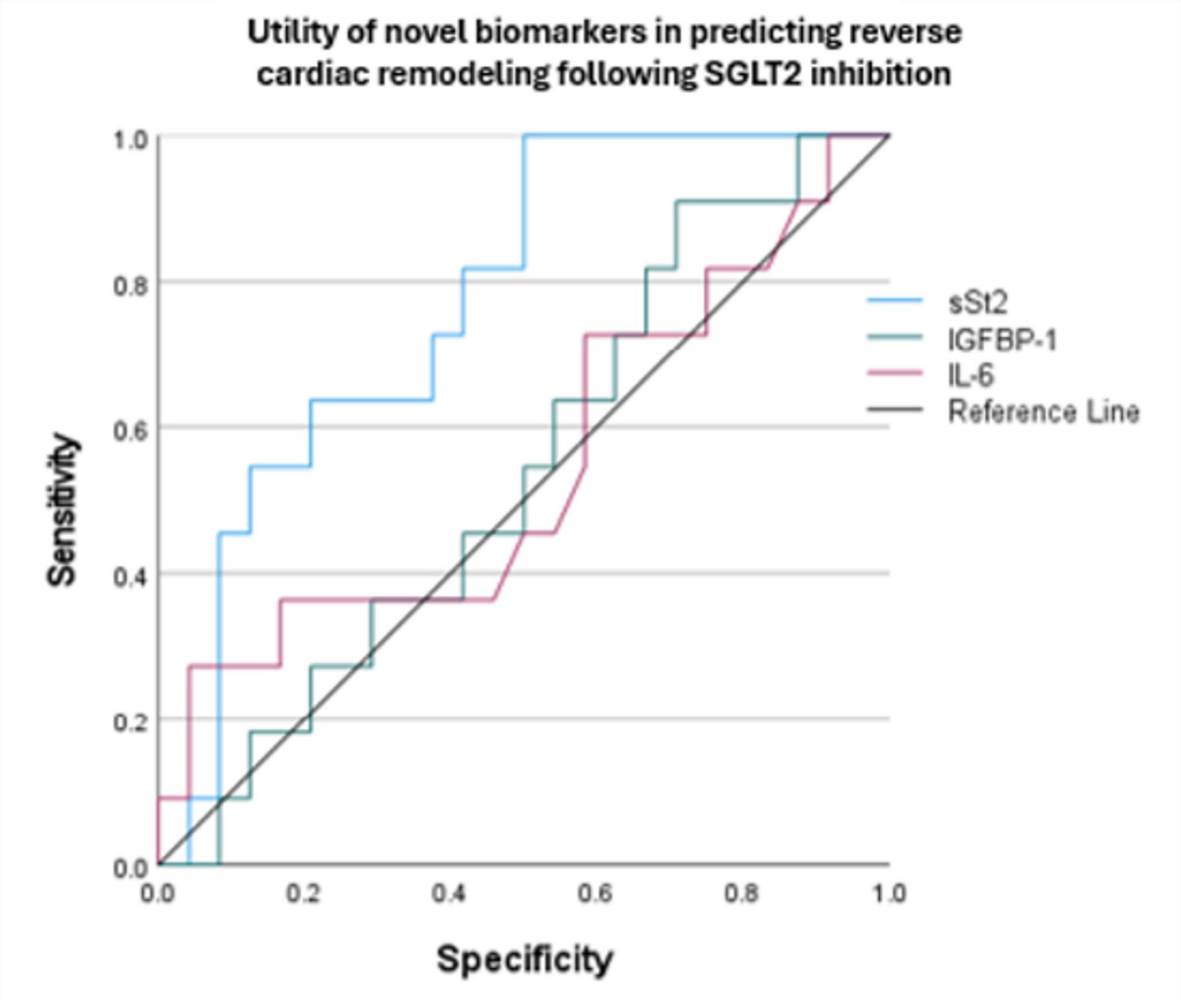
Receiver-operating characteristic (ROC) curve analysis of sSt2, IL-6 and IGFBP-1 and predictive utility of RCR (defined as relative improvement in LV GLS ≥ 10%). Serum sSt2 levels predicted RCR (AUC 0.773; 95% CI 0.62 to 0.96: p = 0.008 with a cut off value of 37ng/L yielding an 83% sensitivity. Both baseline serum IL-6 and IGFBP-1 did not predict RCR (AUC 0.562: 95% CI 0.36 to 0.77: p = 0.10) and (AUC 0.515: 95% CI 0.32 to 0.71: p = 0.88) respectively.AUC: Area under the curve, LV GLS: Left ventricular global longitudinal strain, RCR: Reverse cardiac remodeling, SGLT2i: Sodium-glucose co-transport 2 inhibitor, sST2: soluble suppression of tumorigenicity 2 protein, IL-6: Interleukin-6 and IGFBP-1: Insulin growth factor binding protein

At a median follow-up of 196 days, +RCR was noted in 13 patients (mean relative improvement + 24.0+/−18.3 %). Baseline serum sSt2 levels significantly predicted + RCR (AUC 0.773; 95% CI: 0.62–0.96: *p* = 0.008) with a Youden Index cut-off value of 37.3 ng/L yielding an 83% sensitivity. Both baseline serum IL-6 and IGFBP1 did not predict + RCR (AUC 0.562; 95% CI 0.36–0.77: *p* = 0.10) and (AUC 0.515; 95% CI 0.32–0.71: *p* = 0.88), respectively (Fig. 4).

### Sub-analysis of Novel Biomarker Correlation with LVEF (%)

Of the patients who underwent echocardiographic assessment, 36 had windows sufficient for Simpsons biplane analysis. Although this study was not powered to detect changes in LVEF, when assessing patients with impaired LVEF at baseline (*n* = 29), a significant improvement in LVEF was noted following SGLT2 inhibition (41.8+/−6.7 % to 44.1+/−9.1 %, *p* < 0.001). Interestingly, sSt2 was modestly negatively correlated with improvement in LVEF (*r*= −0.412, *p* = 0.02) (Fig. 3b). Neither IL-6 nor IGFBP-1 were correlated with LVEF improvement (*p* = 0.2) and (*p* = 0.146) respectively.

### Standard Biomarkers

The delta change in standard of care biomarkers in response to SGLT2 inhibition was also assessed (Table 4). A significant increase in haematocrit (41.4+/−3.9 % to 43.7+/−3.9 %, *p* < 0.001) and haemoglobin (14.1+/−1.4 g/dL to 14.8+/−1.4 g/dL, *p* < 0.001) was noted however no significant change in NT-proBNP (+ 174.1 ng/L [95% CI: −38.1 to 33.1; *p* = 0.441]) or HbA1c (+ 4.7 mmol/mol [95% CI: −13.6 to 4.1; *p* = 0.746)] was seen (Fig. 2b). Additionally, no significant correlations were noted between any of the markers tested and change in GLS % following SGLT2i. A full description of these data are available in the appendix (A5).

**Table 4:** Change in standard care biomarkers following six months SGLT2 inhibitor therapy

Change in standard care biomarkers following SGLT2 inhibition					
Standard care biomarkers	Number pairs *(n)*	Baseline	Follow-up	Mean difference (95% CI)	P value
NT-proBNP (ng/L) *	40	845 (135.3 - 920.8) *	912 (122.0 - 1073.8) *	+174.1 (-381.2 to 33.1)	p=0.441
CRP (mg/L)	40	4.4+/-5.4	5.7+/-6.2	+1.4 (-3.7 to 1.0)	p=0.715
Troponin (ng/L)	40	12.2+/-5.8	11.5+/-6.8	-0.7 (-0.5 to 1.9)	p=0.061
HbA1c (mmol/mol)	40	37.2+/-3.8	41.9+/-3.8	+4.7 (-13.6 to 4.1)	p=0.764
Haematocrit (%)	40	41.4+/-3.9	43.7+/-3.9	+2.2 (-2.9 to -1.4)	**p<0.001**
Haemoglobin (g/dL)	40	14.1+/-1.4	14.8+/-1.4	+0.7 (-8.8 to -4.1)	**p<0.001**
Platelets (x10^9/L)	40	224.1+/-85.1	222.4+/-74.6	-1.7 (-22.7 to 26.1)	p=0.826
White cell count (x10^9/L)	40	7.3+/-2.3	7.3+/-2.2	0.0 (-0.7 to 0.7)	p=1.000
Sodium (mmol/L)	40	138.6+/-2.3	139.1+/-2.1	+0.5 (-1.2 to 0.2)	p=0.110
Potassium (mmol/L)	40	4.5+/-0.5	4.4+/-0.3	-0.1 (-4.6 to 6.7)	p=0.151
Creatinine (mmol/L)	40	96.9+/-24.8	97.1+/-26.2	+0.3 (-4.3 to 6.7)	p=0.399

Data presented as mean (SD), *Data presented as mean (IQR). Differences in means assessed using a paired two tailed t-test with significance defined as *p* < 0.05

*CI* Confidence interval, *CRP* C-reactive protein, *HbA1c* glycosylated haemoglobin, *NT-proBNP* NT-pro brain natriuretic peptide, *SGLT2i* Sodium-glucose co-transport 2 inhibitor

### QoL Outcomes

At six months follow-up, KCCQ-12 score improved significantly (43.3+/−13.4 to 52.6+/−10.4, *p* < 0.001). No correlation was noted between delta change in KCCQ-12 score and GLS% (*r* = 0.03, *p* = 0.459) or LVEF % (r-0.001, *p* = 0.934). Additionally, duration of therapy did not appear to influence KCCQ-12 (*r* = 0.113, *p* = 0.487). Furthermore, no correlation was noted between sSt2 (*r* = 0.06, *p* = 0.702), IL-6 (*r* = − 0.06, *p* = 0.692) or IGFBP-1 (*r*=−0.243, *p* = 0.169) and QOL score, nor was any association noted between any standard of care biomarker and symptom improvement as defined by KCCQ-12 (Appendix A6).

## Discussion

SGLT2 inhibitors are now a pillar of guideline directed HF care [2]. Unlike traditional HF therapies they do not appear to interact with the renin-angiotensin system but rather may exert their pluripotent effects via alternative pathways [28, 29]. Indeed, their proven cardio-protective effects, which are not fully explained by their effects on natriuresis, blood pressure and blood glucose alone. Much interest surrounds their potential modulatory effects on inflammatory and anti-fibrotic pathways with implication of specific cytokines such as IL-1β, IL-4 and IL-6 along with the proteins IGFBP-1 and sSt2 [9, 23, 27]. There are limited clinical data evaluating these novel biomarkers in a HF population, in concert with echocardiographic data.

In this study, we have demonstrated that the novel cardiac protein sSt2 is suppressed by SGLT2 inhibition and correlated with improvements in LVEF. Furthermore, baseline sSt2 exhibits a predictive utility to identify which patients treated with SLGT2i will experience improvement in cardiac function, as determined by a ≥ 10% improvement in GLS. These changes remained consistent when delineated by ischaemic versus non-ischaemic aetiology.

The novel protein sSt2, is a member of the IL-1 receptor family and has an important role in mediating inflammatory responses. Its production is stimulated by myocardial stretch with higher levels associated with higher mortality in CVD and HF [26, 30]. It acts by binding and inhibiting the action of IL-33, which normally acts to protect against Ang-2 driven adverse cardiac remodeling. The observation of a possible suppressive effect of SGLT2 inhibition in patients with HF serves as an interesting findings which warrants further exploration, in particular given the recent findings with respect to SGLT2 inhibitors effects on cardiac remodeling [14].

Emerging data has implicated SGLT2 inhibition with suppression of several novel pro-inflammatory proteins, specifically, IL1-β, IL-4 and IL-6 [9, 27]. In our study, we failed to demonstrate SGLT2 inhibition induced significant changes in any of these novel proteins. Additionally, IGFBP-1 (a novel protein implicated in fibrosis), did not change significantly following SGLT2 inhibition. Furthermore, changes in IGFBP-1 levels were not associated with any echocardiographic markers of cardiac remodeling.

Interestingly, although overall IL-6 levels were not significantly affected by SGLT2 inhibition, a modest positive correlation was noted with improvements in GLS. Furthermore, when delineated by aetiology we found that this effect only remained significant in patients with non-ischaemic HF. Additionally, there seemed to be a dichotomous signal with IL-6 trending up in patients with no improvements in GLS and down in those with improvements in GLS. Albeit, this trend was non-significant, in conjunction with the significant correlation data it may serve to highlight a possible signal which may become clearer with a larger sample size or possibly longer duration of therapy. It also highlights the merits of further exploration with distinct HF populations.

IL-6 is a pro-inflammatory cytokine associated with coronary artery disease, insulin resistance and endothelial dysfunction. Animal studies have demonstrated that SGLT2 inhibition promotes HKII and ERK1/2 mediated suppression of IL-6 levels with early observational clinical data re-iterating these findings [31]. Indeed, in a mouse model of HF, chronic activation of IL-6 has been demonstrated to promote LVH with subsequent deletion reversing these effects [32]. This has led to much interest in targeting IL-6 as a therapy in patients with myocardial dysfunction, notably in the recent study, ASSAIL-MI, where administration of tocilizumab (a biologic IL-6 inhibitor) reduced infarct size and improved viable myocardial tissue post STEMI as demonstrated via cardiac MRI [33]. In our study, the observed correlation of IL-6 with LV GLS (%) is potentially hypothesis generating and may warrant further exploration.

Consistent with previous data, we noted significant improvements in both haematocrit and haemoglobin levels following SGLT2 inhibition. Conversely, we did not note significant changes in NT-proBNP, troponin, CRP or WCC. It must be noted that this study was not powered to detect changes in these biomarkers and therefore this lack of effect may merely be reflective of this. Additionally, we saw significant improvement in QoL metrics, however these were not correlated with novel biomarkers or echocardiographic markers of reverse cardiac remodeling. Moreover, despite the significant increases in haemoglobin and haematocrit (which plausibly may improve preload, cardiac output and oxygenation), neither of these markers were correlated with symptom improvement. This finding is reflected in data from the landmark SGLT2i trials such as DAPA-HF and EMPORER-REDUCED, where the symptomatic benefits of SGLT2 inhibition were noted very early (within weeks), before biomarker changes [10, 34]. Therefore, it is plausible that these occur independently to observed echocardiographic or biomarker changes and again, a longer duration of follow-up may facilitate a meaningful change in these parameters.

### Limitations

There are several limitations to this study. Firstly, given it is a prospective observational study it is exposed to selection bias and risk of confounding factors. Furthermore, general interpretation of this data is limited to association of effect and not causation. These data are therefore presented as hypothesis generating only. Secondly, this study was powered to detect changes in IL-6, sSt2 and GLS, and not to detect changes in standard of care biomarkers nor other echocardiographic findings such as LVEF. Thirdly, our follow-up period was limited to six months, which may not be sufficient to fully realise echocardiographic changes secondary to SGLT2 inhibition. Fourthly, our median time from HF diagnosis was over six years therefore the majority of patients in this group had well established HF, with limited scope for reverse cardiac remodeling. It is plausible that this may have limited the potential impact of SGLT2 inhibition on both novel biomarkers and echocardiographic parameters. Finally, the aetiology of our HF population contained both ischaemic and non-ischaemic cardiomyopathies. At a cellular level the respective mechanistic pathways may differ and therefore conclusions drawn from these data should be guarded.

### Summary

Emerging data has implicated SGLT2 inhibitor therapy in HF with modulation of inflammatory and fibrotic pathways. In this study of stable HF patients treated with SGLT2 inhibition, we have demonstrated an inhibitory effect on the novel cardiac protein sSt2 which was modestly correlated with improvements in LVEF. These effects appeared to be independent of symptom improvement and baseline demographics. We did not note an anti-inflammatory effect secondary to SGLT2 inhibition, with no significant changes in novel pro-inflammatory biomarkers of interest, including IL-1β, IL-4, IL-6 and IGFBP-1. These data are novel in the context of current literature and are hypothesis generating. Further understanding of the mechanistic implications of sSt2 in this context are warranted. In particular, evaluation in distinct HF populations (both ischaemic and non-ischaemic respectively) may provide valuable insight.

## Supplementary Information

Below is the link to the electronic supplementary material.

Supplementary Material 1

## Data Availability

Data are available within reasonable request.
