# Supplementary material for: Investigation of Biomarker Response to SGLT2 Inhibition in Heart Failure (SiN-HF)
Source: Cardiovasc Drugs Ther. 2025 Dec 5;40(4):1423–33. doi: 10.1007/s10557-025-07800-3 (PMC13388772; doi:10.1007/s10557-025-07800-3)
Supplement: Supplementary file 1 — Supplementary Material 1 [file 10557_2025_7800_MOESM1_ESM.docx]

**APPENDIX**

1. **Change in clinical parameters following SGLT2 inhibition**

| **Change in clinical parameters following SGLT2 inhibition** | | | | | |
| --- | --- | --- | --- | --- | --- |
|  | | **Baseline** | **Follow-up** | **Mean difference (95% CI)** | **p value** |
| Weight (kg) | | 80.7+/-19.4 | 79.5+/-18.1 | -1.1 (-0.8 to 3.1) | p = 0.245 |
| Blood pressure (mmHg) | |  |  |  |  |
|  | Systolic | 130.6+/-21.6 | 127.4+/-19.9 | +3.1 (-4.5 to 10.8) | p = 0.411 |
|  | Diastolic | 76.8+/-11.7 | 76.6+/-11.6 | -0.2 (-3.7 to 4.1) | p = 0.913 |
| Heart rate (bpm) | | 72.0+/-11.8 | 74.6+/-11.2 | +2.6 (-7.4 to 2.2) | p = 0.276 |
| Saturations (%) | | 95.1+/-2.1 | 95.2+/-2.1 | +0.1 (-1.0 to 0.9) | p = 0.908 |
| KCCQ 12 score | | 42.3+/-13.4 | 52.6+/-10.4 | **+9.3 (-12.6 to -6.1)** | **p<0.001** |
| NYHA score | | 2.4+/-0.5 | 1.6+/-0.6 | **-0.8 (0.6 to 1.0)** | **p<0.001** |

***Table*** demonstrating change in clinical parameters and QoL scores following SGLT2 inhibition. Data displayed as mean+/-SD. Significance defined as p value <0.05.

CI: Confidence interval, KCCQ-12: Kansas City Cardiomyopathy Questionnaire-12, NYHA: New York Heart Association

- 1. **Concurrent cardiac pharmacotherapy**

***Bar chart*** demonstrating cardiac pharmacotherapy at baseline and follow-up in study population.

ACS: Angiotensin converting enzyme inhibitor, ARB: Angiotensin receptor blocker, ARNI: Angiotensin-neprilysin inhibitor, MRA: Mineral corticoid receptor antagonist.

**2.0. Sub-analysis by aetiology (ischaemic vs non-ischaemic)**

**2.1. Demographics**

| **DEMOGRAPHICS DELINEATED BY AETIOLOGY** | | | | |  |
| --- | --- | --- | --- | --- | --- |
|  | | **Ischaemic (n=16)** | **Non-ischaemic (n=24)** | **P value** |  |
| **Age (yrs)** | | 68.1+/-8.5 | 66.6+/-8.2 | 0.570 |  |
| **Gender** | |  |  |  |  |
|  | Male (n, %) | 13 (81.3) | 15 (62.5) | 0.205 |  |
|  | Female (n, %) | 3 (18.8) | 9 (37.5) |  |  |
| **Time on SGLT2i (days)** | | 196 +/-27.9 | 200+/-29.7 | 0.575 |  |
| **Left ventricular ejection fraction (%)** | | 44.5+/-8.3 | 45.8+/-9.7 | 0.691 |  |
| **SBP (mmHg)** | | 131.1+/-22.9 | 127.9+/-21.4 | 0.640 |  |
| **DBP (mmHg)** | | 79.1+/-13.9 | 75.3+/-9.8 | 0.295 |  |
| **Creatinine (mmol)** | | 100.5+/-27.0 | 99.9+/-40.5 | 0.959 |  |
| **Comorbidities (n, %)** | |  |  |  |  |
|  | Hypertension | 9 (56.3) | 13 (54.2) | 0.897 |  |
|  | Chronic kidney disease | 6 (37.5) | 9 (37.5) | 1.000 |  |
|  | Hypercholesterolaemia | 7 (43.8) | 6 (25.0) | 0.215 |  |
|  | Myocardial infarction | 14 (87.5) | 2 (8.3) | **< 0.001** |  |
|  | COPD | 3 (18.8) | 5 (20.8) | 0.872 |  |
|  | Type 2 DM | 1 (6.3) | 1 (4.2) | 0.767 |  |
|  | Atrial fibrillation | 3 (18.8) | 9 (37.5) | 0.205 |  |
| **Table 1:** Demographics of study population delineated by aetiology. Categorical data presented as (n, %) with continuous data presented as mean+/-SD. Significance defined as p<0.05. All p-values are two-sided. | | | | |  |
|  |  |  |  |  |  |
|  |  |  |  |  |  |

**2.2. Changes in standard care biomarkers**

| **Change in standard care biomarkers delineated by aetiology** | | |  |
| --- | --- | --- | --- |
| **Novel biomarkers** | **Mean difference (95% CI)** | **p value** |  |
| **Ischaemic (n = 16)** | | |  |
| Haemoglobin (g/dL) | +6.3 (1.6 to 11.0) | **0.012** |  |
| White cell count (x10^9/L) | +0.2 (-1.3 to 1.6) | 0.885 |  |
| Haematocrit | +2.3 (0.3 to 1.1) | **0.001** |  |
| NT-proBNP (ng/L) * | +114.0 (-124.0 to 352.9) | 0.416 |  |
| CRP (mg/L) | +0.5 (-2.4 to 3.4) | 0.724 |  |
| Troponin (ng/L) | -1.0 (-3.5 to 1.6) | 0.416 |  |
| Creatinine (mmol/L) | -1.0 (-8.4 to 6.6) | 0.779 |  |
| **Non-ischaemic (n=24)** | | |  |
| Haemoglobin (g/dL) | +8.2 (4.4 to 11.9) | **<0.001** |  |
| White cell count (x10^9/L) | -0.1 (-0.5 to 0.8) | 0.702 |  |
| Haematocrit | +2.8 (1.3 to 4.2) | **<0.001** |  |
| NT-proBNP (ng/L) * | +65.6 (-412.9 to 281.8) | 0.285 |  |
| CRP (mg/L) | +0.4 (-3.1 to 3.8) | 0.830 |  |
| Troponin (ng/L) | -1.8 (-3.8 to 0.3) | 0.083 |  |
| Creatinine (mmol/L) | -4.5 (-16.1 to 7.1) | 0.700 |  |
| **Table 2:** Change in standard biomarkers following SGLT2 inhibitor therapy, delineated by aetiology. Differences in means assessed using a paired two tailed t-test with significance defined as p<0.05. SGLT2i: Sodium-glucose co-transport 2 inhibitor, CRP: C-reactive protein, HbA1c: glycosylated haemoglobin, NT-proBNP: NT-pro brain natriuretic peptide. | | |  |
|  |  |  |  |
|  |  |  |  |

**2.3 Novel biomarkers**

| **Change in novel biomarkers following SGLT2i therapy** | | |  |
| --- | --- | --- | --- |
| **Novel biomarkers** | **Mean difference (95% CI)** | **p value** |  |
| **Ischaemic (n=16)** | | |  |
| IGFBP1 (ng/ml) | +3063.2 (-7052.9 to 926.6) | 0.123 |  |
| ST2 (pg/ml) | -33.1 (27.3 to 38.8) | **< 0.001** |  |
| IL1-β (pg/ml) | +0.9 (-2.5 to 0.7) | 0.241 |  |
| IL6 (pg/ml) | -1.0 (-6.9 to 2.1) | 0.274 |  |
| **Non-ischaemic (n=24)** | | |  |
| IGFBP1 (ng/ml) | +3712.5 (-11796.8 to 4371.6) | 0.349 |  |
| ST2 (pg/ml) | -38.4 (34.1 to 42.7) | **< 0.001** |  |
| IL1-β (pg/ml) | +0.2 (-0.6 to 0.2) | 0.242 |  |
| IL6 (pg/ml) | + 1.0 (-2.4 to 0.4) | 0.159 |  |
| **Table 3:** Change in novel biomarkers following SGLT2 inhibitor therapy, delineated by aetiology. Data presented as mean (range) with differences in means assessed using a paired two tailed t-test with significance defined as p<0.05. *An insufficient number of patients in our cohort had detectable levels of IL-4 at follow-up, therefore precluding a paired analysis. SGLT2i: Sodium-glucose co-transport 2 inhibitor, sST2: soluble suppression of tumorigenicity 2 protein, IGFBP1: Insulin-like growth factor-binding protein, IL-1B: Interleukin-1 beta, IL-4: Interleukin-4, IL-6: Interleukin-6. | | |  |
|  |  |  |  |
|  |  |  |  |

**2.4. Correlation of novel biomarkers with reverse cardiac remodeling**

| **Correlation of standard biomarkers with LV GLS (%)** | | |  |
| --- | --- | --- | --- |
| **Novel biomarkers** | ***r*** | **P** |  |
| **Ischaemic** | | |  |
| IGFBP1 (ng/ml) | -0.341 | 0.233 |  |
| ST2 (pg/ml) | 0.299 | 0.299 |  |
| IL1-β (pg/ml) * | -0.427 | 0.399 |  |
| IL6 (pg/ml) | 0.305 | 0.289 |  |
| **Non-ischaemic** | | |  |
| IGFBP1 (ng/ml) | 0.183 | 0.454 |  |
| ST2 (pg/ml) | 0.45 | 0.823 |  |
| IL1-β (pg/ml) * | -0.74 | 0.875 |  |
| IL6 (pg/ml) | **0.507** | **0.019** |  |
| **Table 4:** Correlation of novel biomarkers with delta change in GLS (%), delineated by aetiology. Pearson correlation used to assess normally distributed data and Spearman* where non-normally distributed. Significance defined as p<0.05. All p-values two-sided. | | |  |
|  |  |  |  |
|  |  |  |  |
|  |  |  |  |

**2.5. Echocardiographic parameters**

| **Change in echocardiographic parameters following SGLT2i therapy delineated by aetiology** | | | |  |
| --- | --- | --- | --- | --- |
| **Echo parameter** | | **Mean difference (95% CI)** | **p value** |  |
| **Ischaemic (n=16)** | | | |  |
| Global longitudinal strain (%) |  | -2.5 (-1.5 to 1.1) | 0.692 |  |
| LVEF (%) | | +0.1 (-4.0 to 4.1) | 0.971 |  |
| LVMi (g/m2) | | -0.9 (-9.6 to 7.9) | 0.823 |  |
| LVEDVi (mls/m2) | | +2.8 (-4.9 to 10.5) | 0.33 |  |
| LVESVi (mls/m2) | | +2.5 (-2.8 to 7.8) | 0.444 |  |
| LAVi (mls/m2) | | -1.0 (-3.2 to 1.1) | 0.239 |  |
| **Non-ischaemic (n=24)** | | | |  |
| Global longitudinal strain (%) | | +0.7 (-0.6 to 2.1) | 0.299 |  |
| LVEF (%) | | -1.2 (-4.8 to 2.5) | 0.495 |  |
| LVMi (g/m2) | | -1.2 (-11.3 to 8.9) | 0.801 |  |
| LVEDVi (mls/m2) | | +1.1 (-10.7 to 12.9) | 0.446 |  |
| LVESVi (mls/m2) | | +3.3 (-5.5to 12.1) | 0.849 |  |
| LAVi (mls/m2) | | +1.8 (-1.1 to 4.6) | 0.207 |  |
| **Table 5:** Change in echocardiography parameters following SGLT2 inhibitor therapy, delineated by aetiology. Data presented as mean+/-SD with differences in means assessed using a paired two tailed t-test with significance defined as p<0.05.  CI: Confidence interval, SGLT2i: Sodium-glucose co-transport 2 inhibitor, GLS: Global longitudinal strain, LAVi: Left atrial volume index, LVEF: Left ventricular ejection fraction, LVMI: Left ventricular mass index, LVEDVi: Left ventricular end diastolic volume index, LVESVi: Left ventricular end systolic volume index. | | | |  |
|  |  |  |  |  |
|  |  |  |  |  |

**3.0. ECHOCARDIOGRAPHIC RESULTS**

**3.1. Table demonstrating baseline and follow-up echocardiographic parameters**

| **Echocardiographic parameters** | | | | | |
| --- | --- | --- | --- | --- | --- |
|  | **Number *(n)*** | **Baseline** | **Follow-up** | **Mean difference (95% CI)** | **p value** |
| Global longitudinal strain (%) | 36 | -13.9+/-3.8 | -14.1+/-3.7 | -0.1 (-0.8 to 1.1) | p=0.803 |
| LVEF (%) | 36 | 45.2+/-9.7 | 45.7+/-9.8 | +0.4 (-3.1 to 2.3) | p=0.759 |
| LVMi (g/m2) | 39 | 101.4+/-3.9 | 102.9+/-3.9 | +1.48 (-7.7 to 4.7) | p=0.737 |
| LVEDVi (mls/m2) | 36 | 57.3+/-16.2 | 59.0+/-20.5 | +1.2 (-2.9 to 0.6) | p=0.648 |
| LVESVi (mls/m2) | 36 | 31.9+/-13.6 | 34.9+/-15.4 | +2.9 (-8.5 to 2.6) | p=0.282 |
| LAVi (mls/m2) | 37 | 30.0+/-13.1 | 30.6+/-12.9 | +0.5 ( -0.4 to 2.9) | p=0.754 |
| RAai (mls/m2) | 34 | 8.8+/-2.7 | 9.6+/-4.3 | +0.8 (-2.2 to 0.6) | p=0.248 |
| Rvdi (cm/m2) | 36 | 3.7+/-0.7 | 4.1+/-2.6 | +0.4 (-1.3 to 0.5) | p=0.243 |
| TAPSE (mm) | 30 | 21.4+/-4.2 | 20.5+/-3.9 | -0.9 (-0.7 to 2.5) | p=0.243 |

***Table*** demonstrating change in indexed echocardiographic parameters following SGLT2 inhibition using a paired, two-tailed students t-test with significance defined as p<0.05.

CI: Confidence interval, SGLT2i: Sodium-glucose co-transport 2 inhibitor, GLS: Global longitudinal strain, LAVi: Left atrial volume index, LVEF: Left ventricular ejection fraction, LVMI: Left ventricular mass index, LVEDVi: Left ventricular end diastolic volume index, LVESVi: Left ventricular end systolic volume index, RAai: Right atrial area index, Reddi: Right ventricular diameter index, TAPSE: Tricuspid annular plane systolic excursion

**3.2. Bar chart demonstrating echocardiographic volumetric changes following SGLT2 inhibition.**


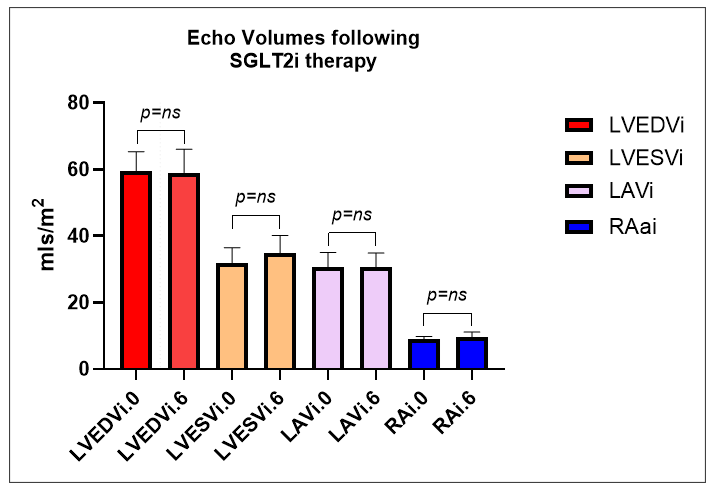


***Bar chart*** demonstrating change in indexed echocardiographic volumetric parameters following SGLT2 inhibition using a paired, two-tailed students t-test with significance defined as p<0.05.

SGLT2i: Sodium-glucose co-transport 2 inhibitor, LAVi: Left atrial volume index, LVEDVi: Left ventricular end diastolic volume index, LVESVi: Left ventricular end systolic volume index, RAai: Right atrial area index.

**3.3. Bar chart demonstrating 2-dimensional echocardiographic changes following SGLT2 inhibition.**


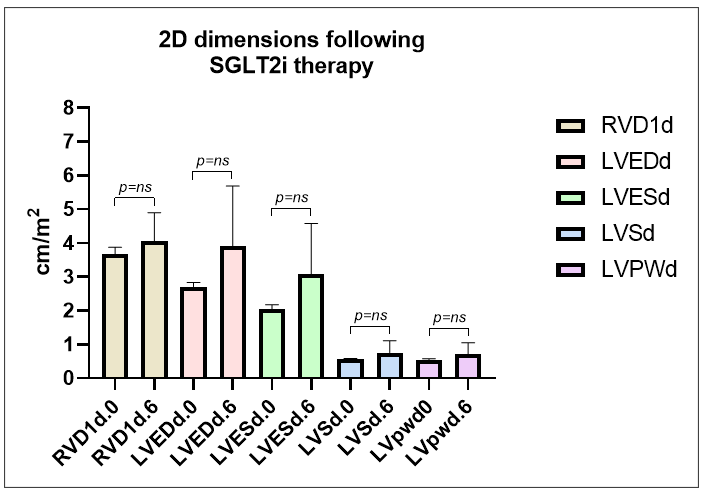


***Bar chart*** demonstrating change in indexed 2-D echocardiographic parameters following SGLT2 inhibition using a paired, two-tailed students t-test with significance defined as p<0.05.

SGLT2i: Sodium-glucose co-transport 2 inhibitor, LVEDdi: Left ventricular end diastolic diameter index, LVESd: Left ventricular end systolic diameter, LVSd: Left ventricle septum diameter, Left ventricle posterior wall diameter.

**3.4. Bar chart demonstrating change in LV Mass following SGLT2 inhibition.**

**
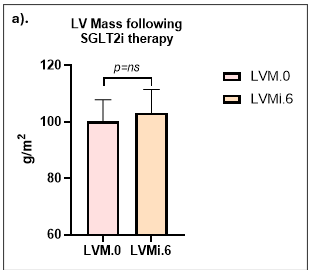
**

***Bar chart* demonstrating** change in indexed LV Mass following SGLT2 inhibition. Analysis performed using a paired, two-tailed students t-test with significance defined as p<0.05. LV Mass calculated using the Devereux formula.

SGLT2i: Sodium-glucose co-transport 2 inhibitor, LVMi: Left ventricle mass indexed.

**3.5. Bar chart demonstrating change in LV global longitudinal strain following SGLT2 inhibition.**

**
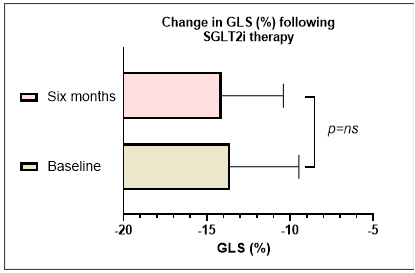
**

***Bar chart* demonstrating** change in GLS (%) following SGLT2 inhibition. Analysis performed using a paired, two-tailed students t-test with significance defined as p<0.05.

SGLT2i: Sodium-glucose co-transport 2 inhibitor, GLS: Global longitudinal strain.

**3.6. Bar chart demonstrating change in LVEF following SGLT2 inhibition.**


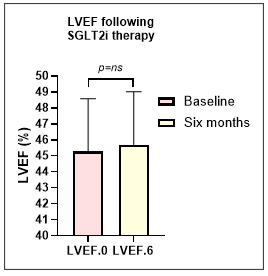


***Bar chart* demonstrating** change in LVEF (%) following SGLT2 inhibition. Analysis performed using a paired, two-tailed students t-test with significance defined as p<0.05.

SGLT2i: Sodium-glucose co-transport 2 inhibitor, LVEF: Left ventricular ejection fraction.

**4.0. SUB-ANALYSIS BY REVERSE CARDIAC REMODELING**

**4.1 Demographics delineated by RCR +/-**

| **DEMOGRAPHICS DELINEATED BY RCR** | | | | |  |
| --- | --- | --- | --- | --- | --- |
|  | | **RCR +ve (n=12)** | **RCR -ve (n=24)** | **P value** |  |
| **Age (yrs)** | | 68.3+/-6.0 | 66.5+/-9.6 | p=0.578 |  |
| **Gender** | |  |  |  |  |
|  | Male (n, %) | 9 (25) | 16 (66.7) | p=0.456 |  |
|  | Female (n, %) | 3 (75) | 8 (33.3) |  |  |
| **Time on SGLT2i (months)** | | 7.0+/-1.0 | 7.0+/-0.9 | p=0.987 |  |
| **Aetiology HF** |  |  |  |  |  |
|  | Ischaemic (n, %) | 5 (41.7) | 9 (37.5) | p=0.809 |  |
|  | Non-ischaemic (n, %) | 7 (58.3) | 15 (62.5) |  |  |
| **Left ventricular ejection fraction (%)** | | 47.2+/-8.8 | 41.9+/-10.8 | **p=0.020** |  |
| **SBP (mmHg)** | | 129.3+/-20.9 | 128.9+/-25.1 | p=0.479 |  |
| **DBP (mmHg)** | | 75.2+/-10.4 | 81.2+/-12.3 | p=0.401 |  |
| **Creatinine (mmol)** | | 93.9+/-25.9 | 111.9+/-49.1 | p=0.278 |  |
| **Comorbidities (n, %)** | |  |  |  |  |
|  | Hypertension | 6 (50.0) | 13 (54.2) | p=0.813 |  |
|  | Chronic kidney disease | 5 (41.7) | 9 (37.5) | p=0.809 |  |
|  | Hypercholesterolaemia | 2 (16.7) | 9 (37.5) | p=0.201 |  |
|  | Myocardial infarction | 4 (33.3) | 9 (37.5) | p=0.806 |  |
|  | COPD | 3 (25.0) | 4 (16.7) | p=0.551 |  |
|  | Type 2 DM | 0 (0.0) | 2 (8.3) | p=0.303 |  |
|  | Atrial fibrillation | 3 (25.0) | 7 (29.2) | p=0.792 |  |
| **Table:** Demographics of study population delineated by +/-RCR. Continuous data presented as mean+/-SD. A X^2^ analysis used to assess differences in categorical variables with a two tailed independent t-test for continuous variables. Significance defined as p<0.05.  COPD: Chronic obstructive pulmonary disease, DBP: Diastolic blood pressure, DM: Diabetes mellitus, HF: Heart failure, RCR: Reverse cardiac remodeling, SGLT2i: Sodium-glucose co-transport 2 inhibitor, SBP: Systolic blood pressure. | | | | |  |
|  |  |  |  |  |  |
|  |  |  |  |  |  |

**4.2. Change in standard biomarkers delineated by RCR +/-**

| **Standard care biomarkers** | | | | | |
| --- | --- | --- | --- | --- | --- |
| **Biomarker** | **RCR-** |  |  |  |  |
|  | ***(n)*** | **Baseline** | **Follow-up** | **Mean dif** | **95% CI (p)** |
| Creatinine (mmol/L) | 24 | 95.5+/-27.9 | 94.9+/-28.3 | -0.6+/-12.1 | [-4.5 to 5.7] p=0.802 |
| Haemoglobin (g/dL) | 24 | 137.5+/-11.8 | 145.7+/-11.9 | **+8.1+/-6.8** | **[5.2 to 11.0] p<0.001** |
| Platelets (x10^9/L) | 24 | 235.5+/-84.1 | 237.2+/-73.2 | +1.8+/-56.5 | [-25.6 to 11.5] p=0.881 |
| Haematocrit (%) | 24 | 40.5+/-3.7 | 43.0+/-3.3 | **+2.5+/-2.2** | **[1.0 to 3.4] p<0.001** |
| MCV (fL) | 24 | 92.2+/-5.0 | 92.0+/-5.9 | -0.2+/-2.9 | [-1.1 to 1.4] p=0.800 |
| WCC (x10^9/L) | 24 | 7.4+/-1.9 | 7.3+/-2.3 | -0.1+/-1.7 | [-3.6 to 1.3] p=0.679 |
| CRP (mg/L) | 24 | 3.3+/-3.8 | 4.5+/-4.5 | +1.2+/-5.8 | [-1.2 to 3.6] p=0.321 |
| Troponin (ng/L) | 24 | 11.0+/-5.7 | 10.2+/-6.7 | -0.8+/-3.9 | [-2.6 to 0.9] p=0.308 |
| NT-proBNP (ng/L) | 24 | 377+/-442.2 | 412+/-519.5 | +35+/-304.5 | [-93.0 to 164.1] p=0.573 |
| HbA1c (mmol/mol) | 24 | 37.4+/-3.3 | 37.4+/-3.4 | 0.0+/-2.2 | [-0.9 to 0.90 p=1.0 |
| Cholesterol (mmol/L) | 24 | 4.6+/-1.2 | 4.5+/-0.9 | -0.1+/-0.8 | [-0.3 to 0.4] p=0.739 |
| **Biomarker** | **RCR+** |  |  |  |  |
|  | **(n)** | **Baseline** | **Follow-up** | **Mean difference** | **95% CI (p)** |
| Creatinine (mmol/L) | 12 | 109.9+/-50.7 | 101.4+/-24.8 | -8.5+/-38.7 | [-33.1 to 16.1] p=0.463 |
| Haemoglobin (g/dL) | 12 | 142.3+/-15.9 | 150.3+/-14.8 | +8.0+/-11.3 | **[0.8 to 15.2] p=0.03** |
| Platelets (x10^9/L) | 12 | 172.6+/-81.2 | 204.1+/-40.3 | +31.4+/-90.6 | [-26.3 to 89.1] p=0.257 |
| Haematocrit (%) | **12** | **41.5+/-4.9** | **44.7+/-4.6** | **+3.2+/-4.4** | **[0.4 to 5.9] p=0.027** |
| MCV (fL) | 12 | 90.5+/-4.5 | 91.5+/-4.1 | +1.0+/-2.8 | [-0.8 to 2.7] p=0.249 |
| WCC (x10^9/L) | 12 | 6.1+/-1.7 | 6.7+/-1.4 | +0.6+/-2.4 | [-1.0 to 2.1] p=0.431 |
| CRP (mg/L) | 12 | 7.8+/-8.7 | 4.7+/-7.3 | -3.0+/-8.7 | [-2.5 to 8.6] p=0.256 |
| Troponin (ng/L) | 12 | 15.1+/-9.2 | 12.4+/-6.5 | -2.7+/-5.9 | [-6.4 to 1.8] p=0.146 |
| NT-proBNP (ng/L) | 12 | 1209+/-1402.1 | 1148+/-1560.8 | -60.9+/-957.1 | [-669.0 to 547.2] p=0.830 |
| HbA1c (mmol/mol) | 12 | 39.4+/-11.0 | 38.5+/-5.4 | -0.8+/-8.6 | [-6.6 to 4.9] p=0.759 |
| Cholesterol (mmol/L) | 12 | 3.5+/-1.2 | 5.9+/-7.3 | +2.5+/-7.4 | [-2.1 to 7.2] p=0.262 |
| **Table:** Within group mean differences in biomarker levels following SGLT2 inhibition in patients with and without RCR, as defined by improvement of GLS ≥10%. Data presented as mean+/-SD. Significance defined as p<0.05. All p-values two-sided.  RCR: Reverse cardiac remodelling, SGLT2i: Sodium-glucose co-transport 2 inhibitor. | | | | | |

**5.0. ECHOCARDIOGRAPHIC CORRELATION ANALYSIS**

**5.1. Standard of care biomarker correlation with LV GLS (%)**

| **Correlation of standard biomarkers with LV GLS (%)** | | |  |
| --- | --- | --- | --- |
| **Biomarker** | ***r*** | **P** |  |
| Haemoglobin | 0.103* | p=0.556 |  |
| Platelets | 0.228 | p=0.145 |  |
| Haematocrit | 0.254* | p=0.469 |  |
| MCV | 0.014 | p=0.671 |  |
| WCC | 0.244 | p=0.231 |  |
| CRP | -0.256 | p=0.081 |  |
| Troponin | -0.081 | p=0.417 |  |
| NT-proBNP | 0.018 | p=0.151 |  |
| HbA1c | 0.101 | p=0.217 |  |
| Creatinine | -0.071 | p=0.913 |  |
| Cholesterol | 0.102 | p=0.764 |  |
| ***Table*** demonstrating correlation of standard biomarkers with delta change in GLS (%). Pearson correlation* used to assess normally distributed data and Spearman where non-normally distributed. Significance defined as p<0.05. All p-values two-sided.  CRP: C-reactive protein, HbA1c: glycosylated haemoglobin, LV: Left ventricle, GLS: Global longitudinal strain, NT-proBNP: NT-pro brain natriuretic peptide, SGLT2i: Sodium-glucose co-transport 2 inhibitor | | |  |
|  |  |  |  |
|  |  |  |  |
|  |  |  |  |

**6.0. QUALITY OF LIFE ANALYSIS**

**6.1. Analysis of effect of time on change in KCCQ-12 score.**

**Figure** demonstrating scatter plot with delta change in KCCQ-12 score plotted against a). LV GLS (%), b). LVEF (%) and c). LV Mass.

Analysis performed using Pearson bivariate regression with two-sided p value. Significance defined as p<0.05.

SGLT2i: Sodium-glucose co-transport 2 inhibitor, GLS: Global longitudinal strain, LVEF: Left ventricular ejection fraction, KCCQ-12: Kansas City Cardiomyopathy Questionnaire-12.

**6.2. Analysis of correlation of echocardiographic changes with change in KCCQ-12.**

**
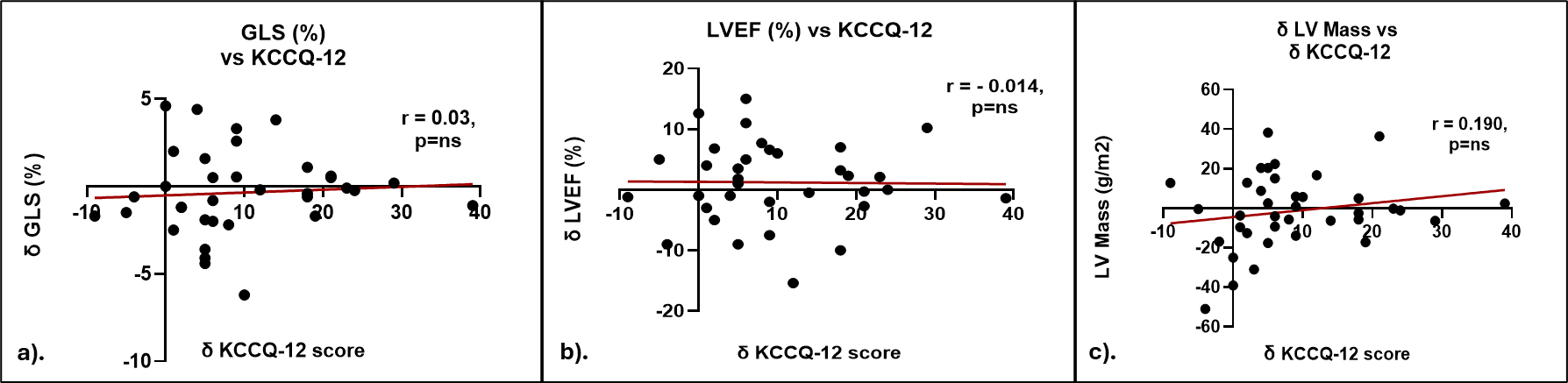
**

**Figure** demonstrating scatter plot with delta change in KCCQ-12 score plotted against a). LV GLS (%), b). LVEF (%) and c). LV Mass.

Analysis performed using Pearson bivariate regression with two-sided p value. Significance defined as p<0.05.

SGLT2i: Sodium-glucose co-transport 2 inhibitor, GLS: Global longitudinal strain, LVEF: Left ventricular ejection fraction, KCCQ-12: Kansas City Cardiomyopathy Questionnaire-12.

**6.3. Novel biomarker correlation with change in KCCQ-12 score.**

**
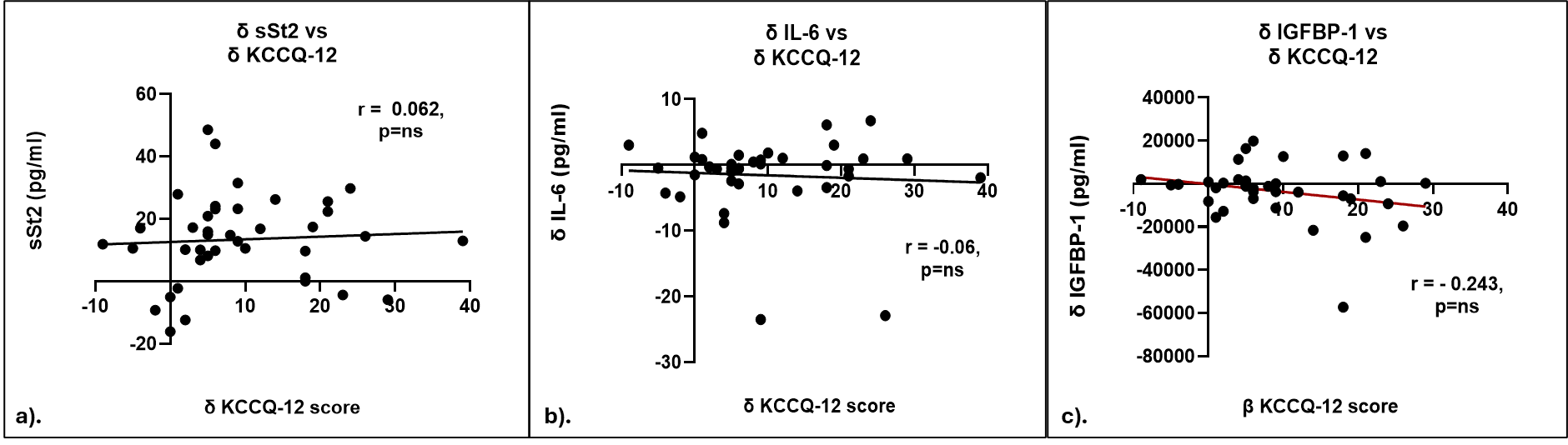
**

**Figure** demonstrating scatter plot with delta change in KCCQ-12 score plotted against a). sSt2 (%), b). IL-6 (%) and c). IGFBP-1.

Analysis performed using Pearson bivariate regression with two-sided p value. Significance defined as p<0.05.

SGLT2i: Sodium-glucose co-transport 2 inhibitor, GLS: Global longitudinal strain, LVEF: Left ventricular ejection fraction, KCCQ-12: Kansas City Cardiomyopathy Questionnaire-12.

**6.5. Standard biomarker correlation with change in KCCQ-12 score.**

**
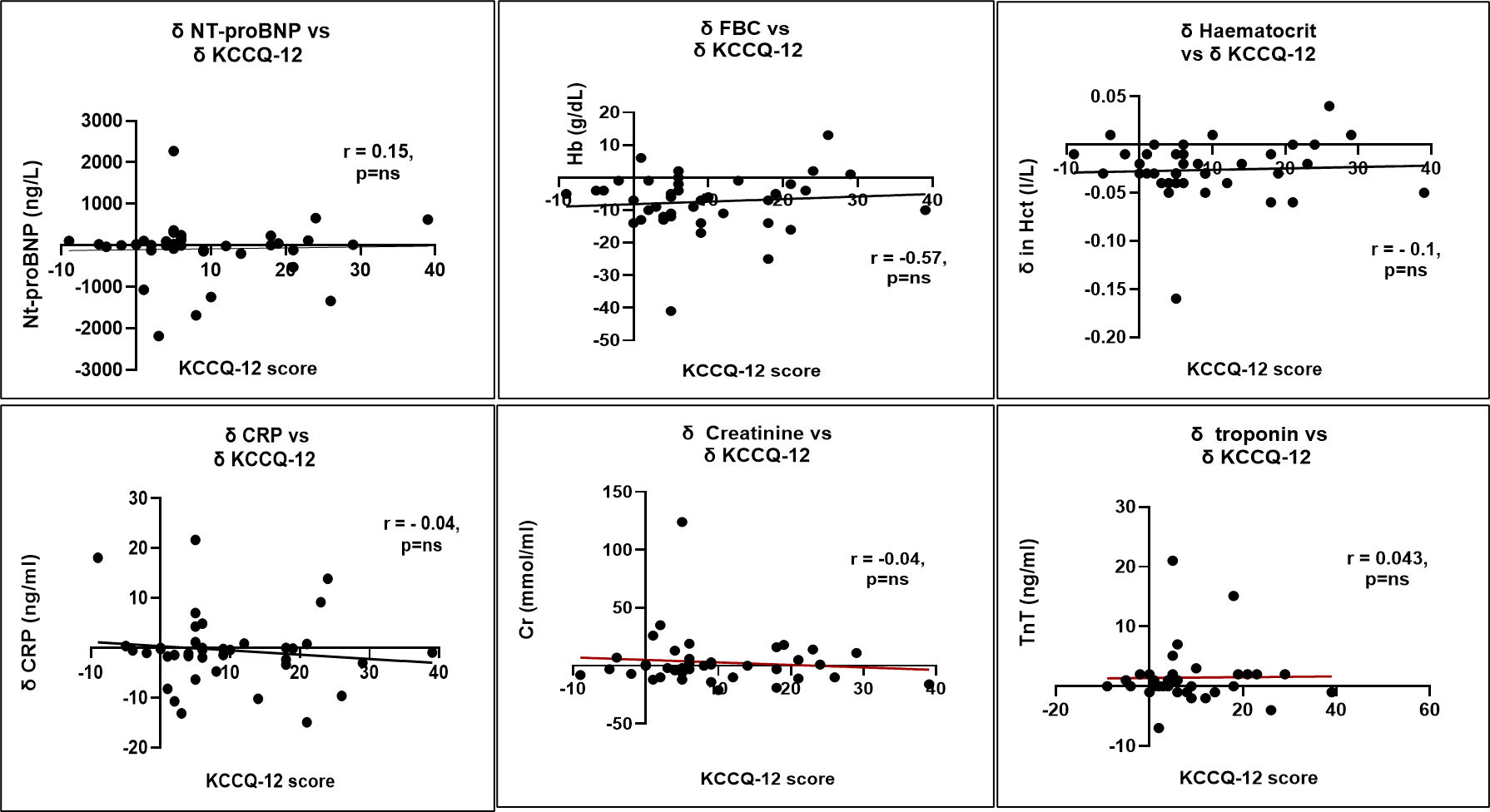
**

**6.6. Standard biomarker correlation with change in KCCQ-12 score** *(continued)*

**Figure** demonstrating scatter plot with delta change in KCCQ-12 score plotted against standard of care biomarkers. Analysis performed using Pearson bivariate regression with two-sided p value. Significance defined as p<0.05.

SGLT2i: Sodium-glucose co-transport 2 inhibitor, GLS: Global longitudinal strain, LVEF: Left ventricular ejection fraction, KCCQ-12: Kansas City Cardiomyopathy Questionnaire-12.
